# Microaerobic dark fermentation by purple bacteria as an emerging perspective for biohydrogen production—a review

**DOI:** 10.1186/s13068-026-02747-5

**Published:** 2026-03-24

**Authors:** S. Krake, G. E. M. Tovar, S. Zibek

**Affiliations:** 1 Fraunhofer Institute for Interfacial Engineering and Biotechnology IGB, Stuttgart, Germany; 2 Institute of Interfacial Process Engineering and Plasma Technology IGVP, University of Stuttgart, Stuttgart, Germany

**Keywords:** Biohydrogen, Microaerobic dark fermentation, Photofermentation, PNS bacteria

## Abstract

While in recent years, a lot of research regarding microbial dark fermentation for biohydrogen production has been carried out focusing on improving well-established processes, this review aims to draw attention to the discovery of a new method of process control, i.e., microaerobic dark fermentation in purple non-sulfur bacteria. This new subsegment of research tries to rethink efficiencies of biohydrogen production and substrate conversion based on an increasingly comprehensive understanding of metabolic pathways, particularly in the area of interplay between aerobic and anaerobic metabolism and the control of this state. By combining hydrogen production through dark fermentation and partially activated photofermentation by a partially active nitrogenase (~ 12%), microaerobic fermentation has the potential to increase hydrogen efficiency. At the same time, it allows to maintain advantages of facultative aerobes, such as high-cell densities and growth rates. Studies show that very high yields of up to 8–12 mol H_2_ mol^−1^ substrate should theoretically be possible with this method, but the yields currently realized are still in the range already achieved with dark fermentation (0.2–1.6 mol H_2_ mol^−1^ substrate). This review provides an overview of the relevant bacteria, metabolism, cultivation, and challenges to further increase hydrogen productivity using microaerobic dark fermentation by purple bacteria. In addition, the model microorganism *Rhodospirillum rubrum* is considered in more detail to gain a deeper understanding of the processes at the metabolic level under microaerobic conditions. The review concludes with a detailed proposal of the challenges and opportunities to create a new, exciting perspective for biotechnological hydrogen production by microaerobic dark fermentation.

## Introduction

The production of hydrogen is a key technology on the road to climate neutrality. There are areas of application that cannot or can hardly be electrified and are dependent on sustainable fuels, such as hydrogen [1]. These include usages, where high power is needed as, e.g., for high-temperature processes in the chemical and steel industries and for shipping or heavy goods transportation in general [2, 3]. For such applications, electric batteries currently cannot provide the energy density (and amount) that is needed. However, hydrogen can not only provide energy but also be an educt for chemical synthesis, e.g., in the production of fertilizers [4]. Here, hydrogen is used to produce ammonium fertilizers. Currently, the Haber–Bosch process is almost exclusively used, but demands extremely high amounts of energy provided by environmentally harmful processes. In addition, there is a growing need for larger amounts of fertilizers to feed the world population, the demand is growing rapidly. While in the year 2020, 90 Mt y^−1^ were needed, projections already indicate a demand of up to 135 Mt y^−1^ in 2030 and 660 Mt y^−1^ in 2050 [5].

To achieve the worldwide production targets for hydrogen, it might prove beneficial to pursue a variety of different approaches and technologies to diversify sustainable and CO_2_-free hydrogen production. Optimal processes for hydrogen production can then be selected based on the conditions on site, depending on the amount and cost of renewable electricity available, the range of waste streams generated, and the degree of independence desired by a company for their industrial processes. For example, in Germany, there will initially be areas that are not directly connected to a hydrogen network, and local hydrogen production options must be considered. Biotechnological hydrogen production from waste residues can be a useful addition here. It could be an opportunity to help close material and energy cycles directly within the company, increasing industrial sustainability. Biohydrogen is particularly suitable here, as production is scalable and hydrogen can be produced from a variety of waste streams. In addition, production can take place locally and run day and night.

**Fig. 1:**
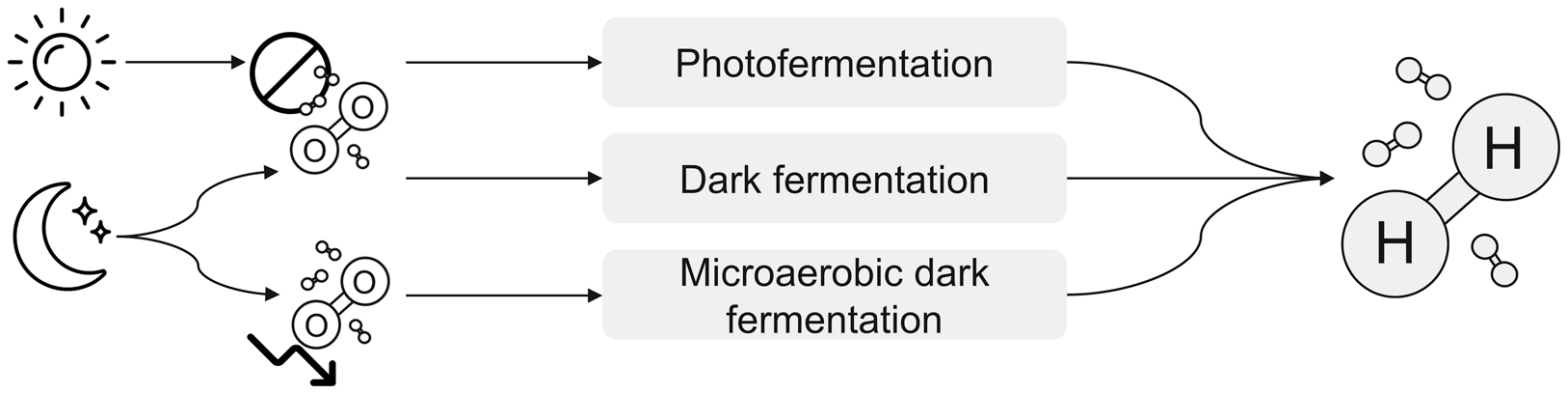
Modes of biotechnological H_2_ production with purple bacteria

There are three production possibilities for biotechnological hydrogen by bacteria (see Fig. 1): anaerobic photofermentation, in which light is absorbed by the bacteria in a similar way to photosynthesis. The light energy is used by the bacteria to fix nitrogen from the air, producing hydrogen as a byproduct and can be conducted photoautotrophically or photoheterotrophically. In anaerobic dark fermentation, sugars are metabolized and excess reduction equivalents are released in the form of hydrogen. Third, microaerobic dark fermentation was recently discovered in purple non-sulfur bacteria, a process that is the subject of this review.

The microaerobic state is defined as an intermediate state between aerobic respiration and anaerobic fermentation at low dissolved oxygen concentrations in which a combination of oxidative and reductive metabolic pathways occurs. Under these conditions, the formation of photosynthetic membranes, which otherwise only occur under photofermentative conditions, can be observed. Under microaerobic, dark conditions, hydrogen can not only be produced by the fermentation of sugars by over-reduction of bacteria, but also by the enzymes of photofermentation [6]. The process benefits from a higher theoretical yield, the use of a wide range of substrates, and the fermentation in simple bioreactors. The process is characterized by the avoidance of high pressures and temperatures and high energy efficiency [7].

This review deals with three PNSB, where microaerobic H_2_ production was already observed during literature research: *Rhodospirillum rubrum*, *Rhodopseudomonas palustris*, and *Rhodobacter capsulatus* [8–10].

## Purple bacteria used for microaerobic hydrogen production

Microaerobic dark fermentation has so far been observed in three strains according to the literature research carried out here (see chapter “cultivation conditions for biotechnological hydrogen production”), which all belong to the phylum *Pseudomonadota *and are summarized under the term purple bacteria. They are gram-negative bacteria, which are characterized by a pink to reddish color. Due to this, they are grouped under the term purple bacteria. This includes all obligate or facultative phototrophic gram-negative proteobacteria. In addition to phototrophic metabolism, facultatives are also capable of aerobic chemotrophic growth. Purple bacteria are subdivided into purple non-sulfur bacteria (PNSB) and purple sulfur bacteria (PSB). Bacteria that store sulfur intracellularly in the form of globules are considered as PSB, while PNSB secrete sulfur extracellularly [11]. The strains *Rhodospirillum*, *Rhodobacter*, and *Rhodopseudomonas* are classified as PNSB, all belonging to the class of *Alpha-proteobacteria.* These bacteria are nowadays mostly found in sewers and waste lagoons, recognizable in the form of a red-pigmented bloom [12].

PNS bacteria have a wide range of applications because of their metabolic diversity as well as a surprisingly high tolerance towards heavy metals during growth make them especially suitable for challenging waste streams and complex substrate matrixes [13, 14]. For these reasons PNSB are researched for bioremediation, i.e., the detoxification of ecosystems, waste water treatment, the production of enzymes and biopolymers, such as polyhydroxybutyrate (PHB) as well as the production of fertilizers for agriculture [15–18]. Especially, in the context of renewable energies, research is currently intensifying, particularly in the direction of microbial fuel cells or fermentative hydrogen production for energy storage.

### *Rhodospirillum rubrum*

*Rhodospirillum rubrum* is a spiral-shaped, 0.8–1 μm large, gram-negative, facultatively phototrophic bacterium that belongs to the family of *Rhodospirillaceae*. The bacterium occurs naturally in aquatic habitats, mostly in freshwater lakes, where it colonizes the area just below the chemocline (thermocline). The chemocline marks the abrupt transition between the oxygen-rich and oxygen-poor water layers. Thanks to its versatile metabolism, *R. rubrum* is perfectly adapted to the constantly changing environmental conditions in its habitat, which, among other things, enables the production of hydrogen [19]. *R. rubrum* is regarded as a model organism in which a special form of microaerobic dark fermentation with particularly high expression of photosynthetic membranes has been observed and studied at the genetic and enzymatic level. The microaerobic growth regiment for hydrogen production occurs at oxygen partial pressure pO_2_ below 0.3% [20]. Since the formation of photosynthetic membranes can be relevant for hydrogen formation, the absorption spectra of the PNSB are of importance. The carotenoids of *R. rubrum* absorb light at 470–550 nm, bacteriochlorophylls at 590 nm and the bacteriochlorophyll reaction center at 882 nm, which can be used to evaluate the formation of photosynthetic membranes. [21].

### *Rhodopseudomonas palustris*

Like other PNSB, *R. palustris* exhibits a rod-like shape and a size around 1–2 µm. It demonstrates an outstanding flexibility between different environmental conditions, which is why it can be found in a huge variety of places, such as sludge, aquatic sediments, ponds, soils and leaf litters [22]. *R. palustris* is a special example of an adaptable metabolism, which stands out even among the purple bacteria. It is part of a rare group of bacteria that can utilize all four metabolic pathways: light-independent pathways, such as chemoheterotrophy, by utilizing carbon from organic compounds, or chemoautotrophy, where energy is obtained from inorganic substances and carbon from CO_2_. On the other hand, the light-dependent metabolic pathways, such as photoautotrophy, in which energy is obtained from light and carbon from CO_2_ and photoheterotrophy, in which light is also used as an energy source and organic substrates are used as a carbon source [23]. The microaerobic growth regiment is observed in *R. palustris* at oxygen concentrations below 3.0% [9]. Compared to *R. rubrum* it is also known for the ability to degrade plant biomass as a carbon source [24]. Carotenoids of *R. palustris* can be measured at 590 nm and bacteriochlorophyll at 805 and 865 nm [25].

### *Rhodobacter capsulatus*

Under optimal growth conditions, *R. capsulatus* is a rod-shaped, 1–6 µm large bacterium. It has a yellow-brownish color and only expresses reddish color when adding malonate or under aerobic cultivation conditions [26]. However, unlike other PNSB, *R. capsulatus* has a special morphological characteristic: the shape of the bacterium varies with the pH of the medium. At a pH less than 7, almost circular bacteria were observed, which tend to form chains; at pH 7, a rod-like shape is recognizable, the length of which increases with increasing pH [26]. Similar to *R. palustris*, the carotenoid of *R.* *capsulatus* absorbs around 590 nm and bacteriochlorophyll α at 805 and 865 nm [27]. Compared to other PNS bacteria, *R. capsulatus* exhibits a microaerobic growth regimen at relatively high pO_2_ below 8.0% [10].

## Cultivation conditions for biotechnological hydrogen production

**Fig. 2:**
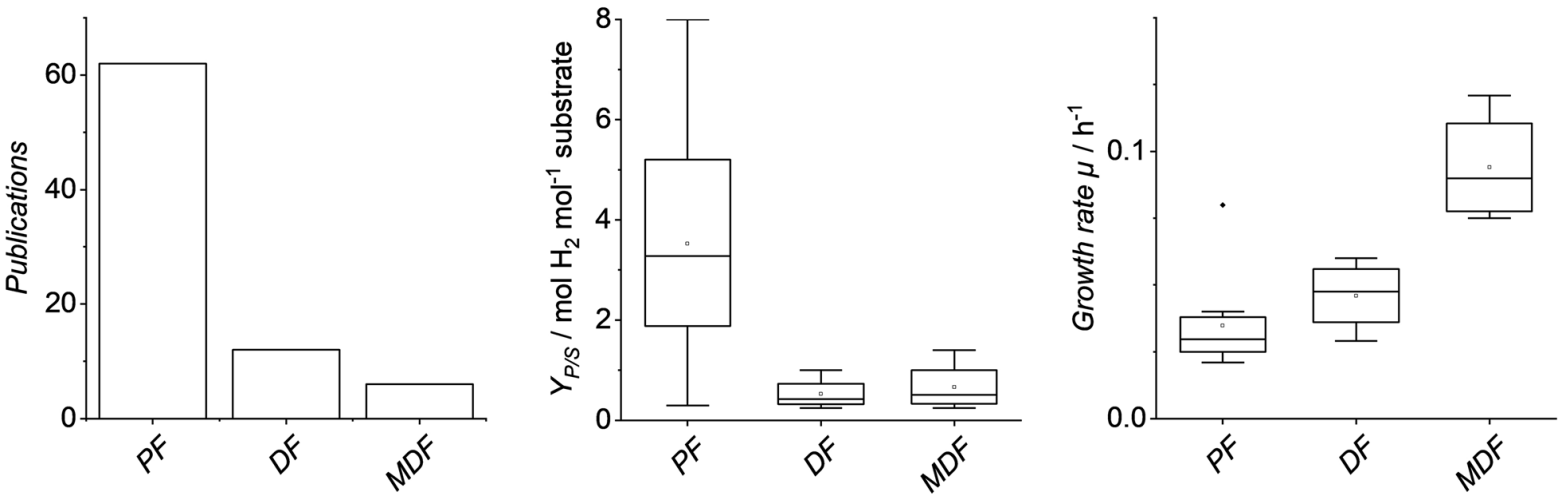
Number of publications describing the fermentation process (left), average H_2_ yield (middle), and growth rate (right) for each biohydrogen process mode (*PF* = photofermentation, *DF* = dark fermentation, *MDF* = microaerobic dark fermentation)

Although there are differences in the metabolic pathways and substrate specificities of the three strains mentioned, they are all currently being investigated for use in biotechnological hydrogen production processes. To provide an overview of the differences in the processes, an extensive literature research was conducted regarding the biotechnological hydrogen production with purple bacteria; an excerpt is shown in Table 1. For data collection, literature research was conducted using the “Web of Science” (WoS) database. The keywords “purple bacteria” and “hydrogen” were chosen within the energy fuels category, excluding hits with “algae”, “food”, “pathways”, “gene”, “enzyme” and “matrix”. The search query was narrowed down to 96 hits, of which 80 were condensed and analyzed in terms of fermentation conditions, substrates, hydrogen yields, and productivity, in addition to analytical tools for fermentation characterization. The results show that photofermentation is, despite the disadvantage in terms of required space, as photobioreactors require an immense amount of space, still the most dominant research field (62 hits), while dark fermentation is focused less (12 hits). Microaerobic dark fermentation is still a fairly new field of research, only present in six publications describing a fermentation process. Cultivation of each type of fermentation is typically conducted at 30 °C and near neutral pH in a batch mode with varying substrates of sugars and organic acids. Most commonly, two nitrogen sources were used: glutamate and ammonium chloride. A trend is observable, where glutamate is routinely used for anaerobic photofermentation as ammonium chloride can inhibit enzymes required for photofermentation and H_2_ evolution [28]. A short comparison of these three modes of operation is given in Fig. 2.

**Table 1:** Literature overview on *R. rubrum*, *R. palustris* and *R. capsulatus* describing microaerobic (bold), dark and photofermentation of H_2_. CSTR = continuous stirred-tank reactor

Bacteria	Strain	Light or dark	Oxygen condition	Temperature [°C]	pH	C-source	N-source	µ [h^−1^]	Y_P/S_ [mol mol^−1^]	Mode	Lit
*R. rubrum*	UR2	Light	Anaerobic	24	6.8	Na-succinate	Na-glutamate	0.02	4.1	Batch	[32]
	ATCC 11170	Dark	Anaerobic	30	7.3	Acetate, CO	NH_4_Cl	0.06	1.0	Fed-batch	[33]
	ATCC 11170	Dark	Microaerobic	30	6.9	Fructose, NH_4_^+^-succinate	NH_4_^+^-succinate	0.10	**0.2**	Batch	[8]
	ATCC 11170	Dark	Microaerobic	30	6.9	Fructose, NH_4_^+^-succinate	NH_4_^+^-succinate	0.10	–	Batch	[19]
*R. palustris*	420L	Light	Anaerobic	28	6.9	Malate	Na-glutamate	0.02	2.9	Fed-batch	[34]
	GCA009	Dark	Microaerobic	30	6.8	Na-lactate	Na-glutamate	–	**1.4**	Batch	[9]
	DSM 127	Light	Anaerobic	30	6.8	Acetate, Butyrate, Lactate, Malate	Ammonium acetate	0.04	1.6	Batch	[35]
	WP3–5	Light	Anaerobic	37	7.0	Sucrose	NH_4_Cl	–	6.1	Batch	[36]
	WP3–5	Light	Anaerobic	37	6.5	Formate, Acetate, Lactate, Butyrate, Ethanol	Yeast extract	0.08	3.0	CSTR	[37]
*R. capsulatus*	JP91	Dark	Microaerobic	30	6.8	Succinate, Lactate, Acetate, Malate	Na-glutamate	–	**0.6**	Batch	[10]
	B10	Dark and Light	Anaerobic	28	6.8	Starch, Butyrate	NH_4_Cl	0.09	1.1	Batch	[38]
	JP91	Light	Anaerobic	30	6.9	Glucose	Na-glutamate	0.02	4.0	CSTR	[39]
	DSM 1710	Light	Anaerobic	30	7.4	Sugar beet molasses	Sugar beet molasses	0.04	6.2	Batch	[40]

Photofermentation achieves the highest molar yield, since the photoreaction is only indirectly required to maintain cell metabolism, while anaerobic and microaerobic dark fermentation utilize the sugar to a larger extent for bacterial growth and the production of organic acids. With this form of evaluation, it should also be noted that the theoretical molar yield for oligosaccharides can of course be significantly higher than for monosaccharides such as fructose influencing the comparison (in Fig. 2 this was accounted for by recalculating yield down to H_2_ per monosaccharide). Both photofermentation and dark fermentation exhibit a mixed growth-associated product formation of H_2_. By modeling the hydrogen formation, a dominance of the growth-associated kinetic was observed, while non-growth-associated product formation only represents a small part [29, 30]. Most H_2_ evolution occurs during the exponential growth phase and only a low amount during the stationary phase.

While photofermentation may result in the most efficient substrate conversion to H_2_ microaerobic dark fermentation can benefit from higher growth rates and cell densities then both anaerobic process regiments resulting in a higher H_2_ productivity [8, 31]. The substrates used in either photofermentation, dark fermentation or microaerobic conditions are listed in Table 2. Most commonly sugars such as fructose and glucose or organic acids such as acetate, butyrate, propionate and succinate were used. While most PNSB can metabolize glucose, no activity of the Entner–Doudoroff pathway could be observed in *R. rubrum* [20]. Not all substrates can be equally converted in all fermentation regiments. For example, for dark fermentation of *R. rubrum* it is reported that acetate assimilation only occurs in the presence of a C_4_-dicarboxylic acids or CO_2_ [41]. If CO_2_ assimilation by the respective enzymes is active during microaerobic conditions remains part of a scientific discussion [15, 20].

**Table 2:** Substrates used for biohydrogen production with purple bacteria reported in publications. ? = proposed substrate utilization [42]. * Oxidation state of carbon, averaged on the number of carbons in the substrate

Substrate	*R. rubrum*	*R. palustris*	*R. capsulatus*	Oxidation state*	Source
Glucose		✓	✓	0	[39, 43]
Fructose	✓	✓	✓	0	[8, 44, 45]
Lactose			✓	0	[46]
Starch			✓	0	[38]
Hexose			✓	0	[47]
Sucrose		✓	✓	0	[36, 48, 49]
Acetate	✓	✓	✓	0	[33, 50, 51]
Butyrate	✓	✓	✓	− 1	[38, 46, 52]
Lactate	✓	✓	✓	0	[35, 38, 46]
Malate	?	✓	?	+ 1	[34]
Succinate	✓	✓	?	+ 0.5	[8, 53]
Formate		✓		+ 1	[37, 54]
Propionate	✓	✓	✓	− 0.7	[46, 55, 56]
Ethanol		✓	✓	− 2	[37, 57]
Glycerol	✓		✓	− 0.7	[58]

For the microaerobic fermentation regime, only hydrogen evolution activity with fructose, glucose, succinate, lactate, acetate, and malate has been reported. Noticeably, only neutral or positive mean oxidation states of carbons in the substrate were used for microaerobic hydrogen formation. In contrast to this, research for dark fermentation or photofermentation suggests rather reduced substrates, such as ethanol, butyrate, or propionate [59, 60].

## The central enzymes for hydrogen production: formate hydrogen lyase and nitrogenase

The dark fermentation pathway for hydrogen production can be different depending on the habitat the bacteria populates and is generally differentiated if the bacteria are an obligate or facultative anaerobe. Obligate anaerobes use the pyruvate ferredoxin oxidoreductase pathway (PFOR), whereby pyruvate is directly metabolized to acetyl-CoA, CO_2_, and a reduction of ferredoxin. In a reduced state, ferredoxin can then be further used by a hydrogenase to produce hydrogen using the released electrons during the reoxidation of ferredoxin [61]. In facultative anaerobes, such as PNSB, the production of hydrogen is conducted via the breakdown of sugars by pyruvate formate lyase (PFL) and the resulting formate by the formate hydrogen lyase (FHL) [62, 63]. This process is summarized as the PFL pathway. FHL is active under microaerobic and anaerobic conditions in the dark, as under aerobic conditions the more energy-efficient oxidative pathways are preferred. The PFL pathway is a bacterial response to release an excess of electrons, producing hydrogen and organic acids as byproducts. Under aerobic and anaerobic conditions, PNSB metabolizes the organic carbon source, for example, fructose, via glycolysis to the C_3_-carboxylic acid pyruvate. Under the selected cultivation conditions, anaerobic in the dark, pyruvate is converted to formate and acetyl-CoA by the pyruvate formate lyase. As the tricarboxylic acid (TCA) cycle is partly or completely inactive, acetyl-CoA accumulates and is then secreted extracellularly in the form of acetate as a byproduct. Subsequently, formate is metabolized to CO_2_ and H_2_ by FHL in a two-step process. Formate hydrogen lyase is an enzyme complex consisting of the two enzymes formate dehydrogenase (FDH) and [NiFe]-hydrogenase ([NiFe]H_2_ase) [64, 65]:

$$HCOOH\overset {FDH} \longleftrightarrow CO_{2} + 2H^{ + } + 2e^{ - } \overset {\left[ {NiFe} \right] - hydrogenase} \longleftrightarrow H_{2} + CO_{2} \,\Delta G^{0} = \, + 1,3\,\,kJ\,mol^{ - 1}$$

Dark fermentation can, therefore, be summarized in the following reaction equation [66]:

$$C_{6} H_{12} O_{6} + 2H_{2} O\mathop{\longrightarrow}\limits^{FHL}2CH_{3} COOH + 2CO_{2} + 4H_{2} \,\Delta G^{0} = - 206\,kJ\,mol^{ - 1}$$

According to the stoichiometric equation a 100% conversion of substrate to hydrogen would result in the maximum possible yield of 4 mol H_2_ mol^−1^ glucose, but in practice it is often lower (0.2–2 mol mol^−1^, see Table 1) because of overflow metabolism under anaerobic conditions, where parts of the sugar are metabolized to organic acids like formate, succinate or propionate or biopolymers, such as PHB [15].

The nitrogenase is the key enzyme for photofermentative hydrogen production. Its main function is the fixation of nitrogen and converting it into the bioavailable ammonium for bacterial growth, releasing hydrogen as a byproduct. This process is highly energy-consuming, because it is only active under lighting. Under exposure to light, the synthesis of the photosynthetic apparatus and the formation of specialized photosynthetic membranes, as well as the enzyme nitrogenase, are triggered [31, 67]:

$$N_{2} + 8H^{ + } + 8e^{ - } + 16ATP\mathop{\longrightarrow}\limits^{Nitrogenase}2NH_{3} + \, H_{2} + 16ADP + 16P_{i} \, \Delta G^{0} = + 274\,kJ\,mol^{ - 1}$$

As a part of the electron transport chain, the photosynthetic apparatus can absorb near-infrared light and transfer the electrons to Ubiquinon. As a result, by Ubiquinon oxidation, a deprotonization occurs and a proton gradient forms, which induces adenosine triphosphate (ATP) production through ATP synthase. Through these steps, purple bacteria are capable of generating enough energy to maintain nitrogenase activity.

**Fig. 3:**
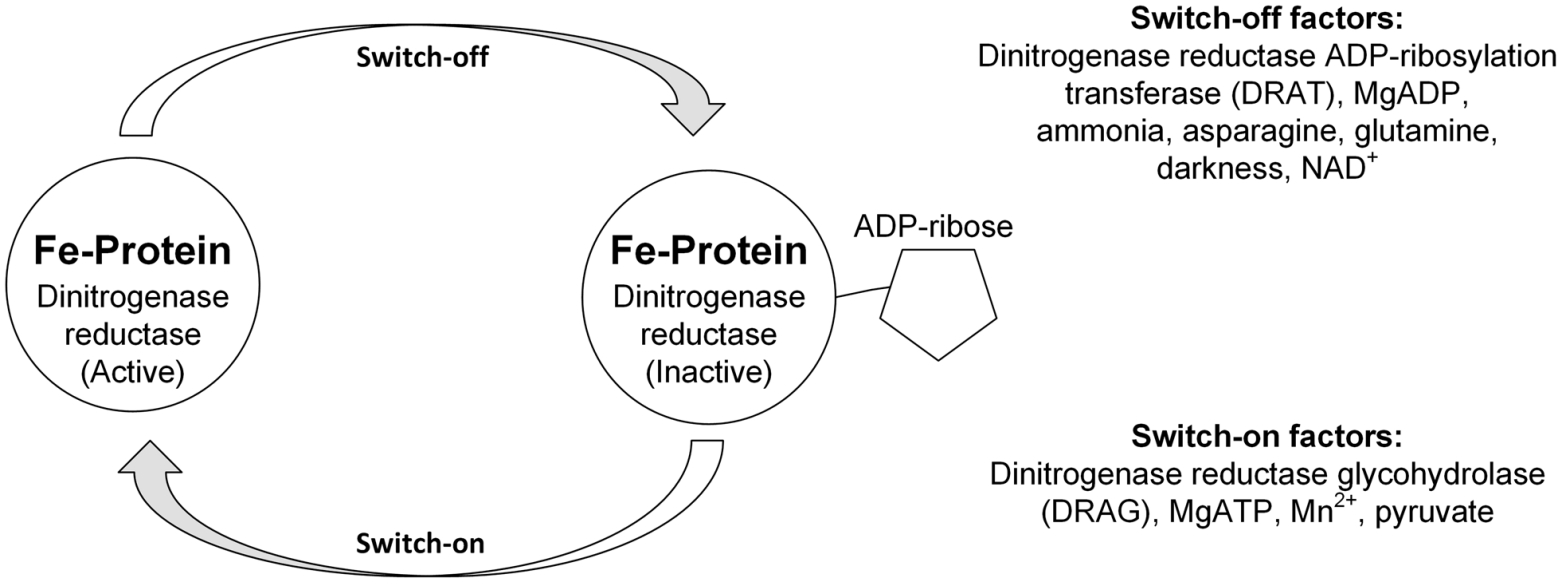
How switch-off and -on factors influence the activity of the Fe-Protein of nitrogenase by ADP-ribosylation [6, 28, 69]

While ATP generation is a key factor for nitrogenase activity, other so-called switch-off or -on factors are highly relevant as well. The switch-off effect describes the sudden change in nitrogenase activity based on changes in environmental conditions downregulating the activity by ADP-ribosylation of the Fe-protein of the nitrogenase by dinitrogenase reductase ADP-ribosylation transferase (DRAT) (see Fig. 3). One factor to evaluate the DRAT regulation is the uridylylation status of the P_II_ proteins. Uridylylation status changes by concentration of cellular metabolites, such as ATP/ADP ratio (uridylylation and deuridylylation), glutamine (deuridylylation), or oxoglutarate (uridylylation) [6, 68]. Uridylylation of the protein results in an increased activity of nitrogenase. This process is reversible if the environmental conditions favor photofermentation.

Besides the uridylylation of proteins, the factors for switch-off and switch-on can be defined by different categories. Some factors, such as ATP and NADH, account for the general energy and reduction state of the cell; others are substrate-related, such as nitrogen sources, trace elements, or pyruvate. Finally, nitrogenases are highly labile, only expressing activity below an oxygen concentration of 1.5% in the light in most cases [70].

## Microaerobic dark fermentation—a promising third pathway

Recently a new field of fermentative hydrogen production emerged, where microaerobic conditions are examined for their potential. The hypothesis is that dark fermentation is a strictly anaerobic process which limits the ability of complete substrate conversion because of a lack of activity of the TCA cycle and oxidative phosphorylation, which is already apparent in the PFL pathway, where acetate is formed from glucose, but further metabolism is impossible, resulting in an extracellular secretion and, therefore, a decrease in possible yield. Only up to a third of the theoretical maximal conversion (4 mol H_2_ mol^−1^ glucose) can be achieved as substrate conversion is accompanied by overflow metabolism and organic acid production which makes dark fermentation inefficient [71]. In addition, the bacteria are unable to effectively use organic acids which limits the use of waste streams from the food or agricultural industry [9]. Photofermentation is an exceptionally efficient conversion to H_2_ compared to dark fermentation but requires a complex lighting concept and thus has a great space requirement on an industrial scale [72]. A microaerobic cultivation regiment can solve both problems as oxidative phosphorylation, and the TCA cycle is still partly oxidative active at these low oxygen concentrations. This enables a reduction of overflow metabolism, an increase of substrate conversion rates as well as a use of organic acids as substrates for hydrogen production, while no light is required [20].

**Fig. 4:**
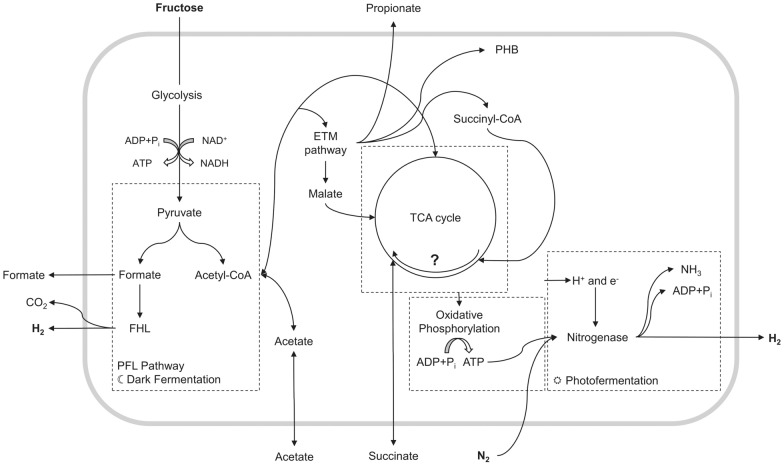
Proposed simplified pathway how oxidative phosphorylation can generate ATP for nitrogenase activity and produce hydrogen by two pathways as well as PHB [10, 74]

An activity of the TCA cycle enables an increased substrate degradation efficiency, which results in a higher rate of NADH generation through substrate oxidation. This results in an excess production of reducing equivalents, which are converted into hydrogen. In addition, NADH is a central substrate in the aerobic respiratory chain, which increases the proton gradient at the membrane and, therefore, increases the production of ATP. These additional amounts of ATP can be used to retrigger the activity of the hydrogen-producing enzyme nitrogenase, at least to a certain extent (~ 12%) [6, 73]. Despite normally lacking the required energy (ATP and NADH) under dark conditions and being inactive by the switch-off effect, a photofermentative H_2_ production can be realized by microaerobic dark fermentation (Fig. 4) [9]. This could possibly lead to hydrogen yields above the theoretical yield of dark fermentation alone.

In addition, to a partly active nitrogenase, hydrogen can be produced by the PFL pathway via FHL as well if conditions regarding oxygen partial pressure and over-reduction of the cells are met. A degree of over-reduction necessary for the formation of photosynthetic membranes and an increased H_2_ yield has so far been achieved by setting microaerobic conditions and using a two-substrate system. The glycolysis of sugar in combination with the partially reductive metabolism of organic acids (in this case, succinate) by the TCA cycle leads to such a reduced state of the bacteria [8]. Instead of a complete excretion of acetate as a final product of the PFL pathway as a result of incomplete substrate conversion, under microaerobic conditions, acetyl-CoA can be further metabolized by the ethylmalonyl pathway (ETM). This results in byproducts, such as propionate or PHB, but also in anaplerotic reactions, where malate and succinyl-CoA can be incorporated into the TCA cycle, increasing substrate conversion efficiency (Fig. 4) [15].

The question thus arises why not use a completely aerobic process to begin with. The reason, as mentioned above, is the extreme oxygen sensitivity of the hydrogen-producing enzymes formate hydrogen lyase and nitrogenase. Therefore, the challenge is to precisely control the microaerobic state and find the optimal compromise between increasing the activity of oxidative phosphorylation while accounting for the sensitivity of hydrogen-producing enzymes to O_2_ to maximize hydrogen productivity under dark conditions. As a result, purple bacteria can produce hydrogen by dark and photofermentation in the dark simultaneously, further increasing substrate conversion rate and hydrogen yield, giving it possibly an advantage over other cultivation regiments. Microaerobic dark fermentation has a combined theoretical maximum yield of 4 mol H_2_ mol^−1^ fructose by dark fermentation and a maximum possible yield of 8 mol H_2_ mol^−1^ fructose by photofermentation at full substrate utilization. In reality, the potential yield is not as high, because comparable nitrogenase activity as in photofermentation could not yet be achieved, and complete substrate conversion does not occur, as can be seen from the accumulation of organic acids during microaerobic fermentation, especially in the form of acetate, but also others, such as propionate, formate, succinate, or malate [15, 19]. A novel approach that does not directly solve this issue, but offers a highly interesting solution to the yields below expectations, is the combination of 3 days of microaerobic dark fermentation followed by a photofermentation phase, as done in small and later also larger scale by Sagir et al. Scaling such a process comes at a price, where simplicity and the low space requirement of a completely dark process will be lost, but by a subsequent conversion of the organic acids formed during microaerobic dark fermentation by a photofermentation phase, exceptionally high yields could be reached (up to 7.8 mol H_2_ mol^−1^ glucose), which is even competitive with the best-studied biohydrogen producers and highly promising for industrial application [74, 75].

## *Rhodospirillum rubrum* as a model organism for microaerobic dark fermentation

**Fig. 5:**
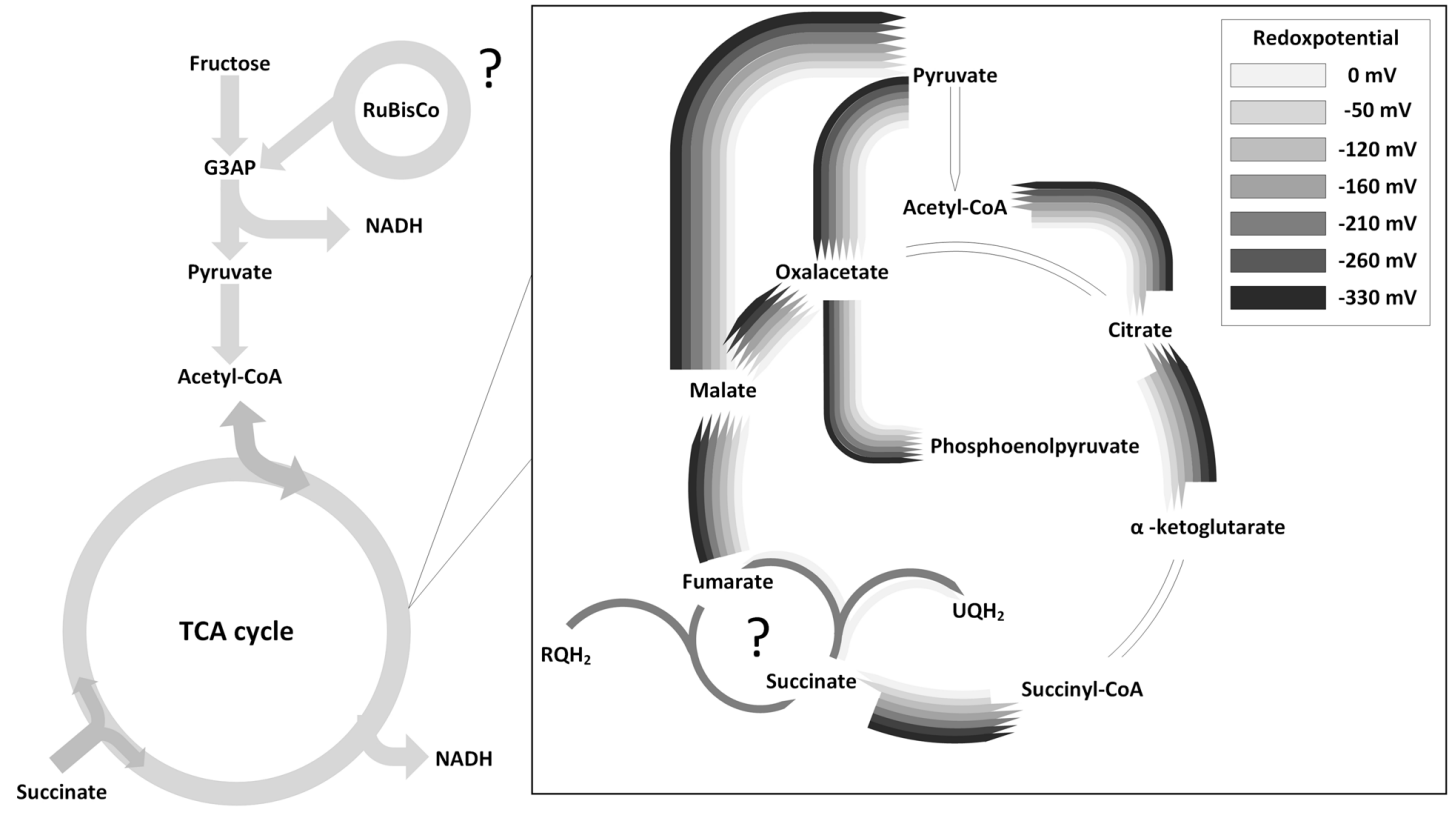
Overview of metabolism pathway of *R. rubrum* under varying oxygen conditions showing the transition between an aerobic and microaerobic fermentation regiment. Arrows indicate the direction of the enzymatic reaction. Created from data of Carius et al. and Grammel et al. [19, 20]

A closer look at the processes behind microbial microaerobic dark fermentation has been made possible by intensive research into the mechanisms of the model organism *R. rubrum* in recent years. This effect is induced only under dark and microaerobic conditions (pO_2_ ≤ 0.3%) with two substrates, one substrate for glycolysis (e.g., fructose) and one for the TCA cycle (e.g., succinate) (see Fig. 5) [20]. The effects of the microaerobic state on the direction of the TCA cycle, the formation of signaling substances to initiate the formation of photosynthetic membranes and, therefore, nitrogenase, and the formation of hydrogen could be shown on a metabolic level by measuring enzymatic activity and apply experimental data to a metabolism model [19, 20].

Glucose metabolism by the Entner–Doudoroff pathway could not yet be observed, showing no activity of G6PDH or KPDG-aldolase [20]. Under dark, microaerobic conditions, an increased activity of the Embden–Meyerhof–Parnas (EMP) pathway can be observed, whereby fructose is metabolized, while the TCA cycle still remains partly active (indicated by an activity of α-KGDH of 29% and 18% of SDH compared to the aerobic activity). Grammel et al. suggested that a partly active, oxidative TCA cycle is a key characteristic of microaerobic dark fermentation. This results in a number of consequences: first, an increase of NADH/NAD^+^ ratio occurs because of an increasing activity of glyceraldehyde 3-phosphate dehydrogenase as well as an isocitrate dehydrogenase activity. At the same time, the activity of TCA is decreased, creating a bottleneck. This leads to the production of hydrogen by formate hydrogen lyase via the dark fermentation pathway, as well as organic acids, such as acetate and formate by the PFL pathway and propionate by the ETM pathway. If the Calvin cycle in the EMP pathway is still active is still unclear and remains a point of discussion. While Grammel et al. could not detect any activity under dark conditions, Godoy et al. and Narancic et al. could measure an activity of ribulose bisphosphate carboxylase (RuBisCo), which represents a central part of the cycle [15, 20, 41]. An active Calvin cycle could compete with the activity of nitrogenase, since CO_2_ assimilation is very energy-consuming in the form of ATP, which is then missing as a reactant in the nitrogenase reaction, decreasing H_2_ productivity [60]. In the partly active TCA cycle, a second substrate, such as succinate but possibly others, such as lactic acid, malic acid, or ethanol, is introduced. An effect is observable that is currently only documented for *R. rubrum*: the succinate dehydrogenase starts to act partly reductive under microaerobic conditions, forming a local reductive cycle between succinate dehydrogenase and fumarate hydratase. This cycle can be seen as the engine of the microaerobic dark fermentation: an increasing amount of Rhodoquinon (RQH_2_) is reduced to Ubiquinol (QH_2_) by succinate dehydrogenase. Thereby fulfilling two conditions for membrane synthesis: a high NADH/NAD^+^ ratio provided by the activity of glyceraldehyde 3-phosphate dehydrogenase, isocitrate dehydrogenase, and a reduced activity of NADH dehydrogenase in the electron transport chain, as well as a high amount of reduced Ubiquinol, which is referred to as an over-reduced state of the cell. These two ratios are the central signal inducers for the expression of photosynthetic membranes and the respective enzymes, especially nitrogenase, enabling H_2_ production by the two pathways and resulting in a purple coloring of the bacteria. In fact, the same levels of signal inducers are achieved as in anaerobic photofermentation [76].

The partly oxidative and reductive activity that was observed by Grammel et al. was confirmed by Carius et al. by a stepwise reduction of the culture redox potential (0 to − 330 mV) by oxygen limitation. The switch to the microaerobic fermentation regiment was measured between − 120 mV and − 160 mV, where α-KGDH switched from an oxidative activity to an increasingly reductive (see Fig. 5). Unlike postulated by Grammel et al., Carius et al. observed that the TCA cycle does not drift continuously between an oxidative and a partially reductive direction in the microaerobic fermentation regiment but forms stable, partly reductive and oxidative TCA cycle operation modes, depending on the applied redox potential (As indicated by the direction of arrows in Fig. 5). The splitting point of the TCA cycle under microaerobic conditions is located in place of the succinate, where the metabolic pathway can proceed in both directions, which could explain the necessity of succinate in the M2SF medium to reach the over-reduced state under microaerobic conditions by a high level of QH_2_ and NADH. Reduced QH_2_ is generated by metabolism of succinate provided as a fermentation substrate by succinate dehydrogenase. The endpoint of microaerobic cultivation was defined below − 330 mV as no uptake of succinate could be measured, therefore, indicating that no succinate oxidation takes place and a fully anaerobic fermentation regiment was reached.

The microaerobic dark fermentation state opens an exciting new perspective, as the enzyme nitrogenase could now also potentially produce hydrogen by the photofermentative pathway enabling two hydrogen pathways without the common limitations, such as a completely anaerobic process or an elaborate lighting system. However, there is still a hurdle to overcome in research before this can be exploited: the switch-off effect of the nitrogenase. While certain conditions are met, such as a sufficiently reduced state or the oxygen lability, other factors for nitrogenase activity need to be addressed, such as dark conditions or the presence of ammonium. Ludden et al. could already achieve a nitrogenase activity of 12% with *R. rubrum* in the dark and a microaerobic state by addition of pyruvate, which acts as an activity promoter; this was further confirmed by Selao et al. [6, 73].

**Fig. 6:**
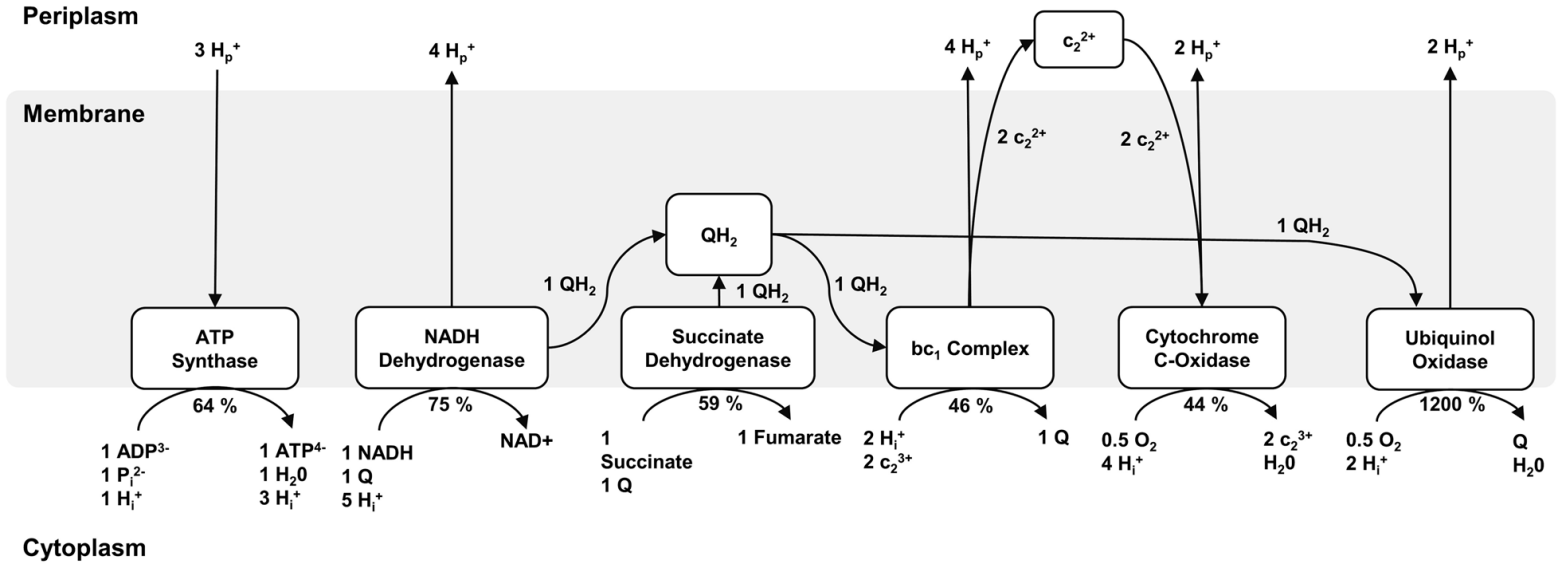
Electron transport chain of *R. rubrum* during microaerobic dark fermentation. As no activity of the reaction complex could be detected under microaerobic conditions, it was not included in the figure. Q = Ubiquinon, QH_2_ = Ubiquinol. Enzyme activities in relation to their aerobic activity in percent are indicated below the enzymes, processes proposed by Klamt et al. [77]

To further understand why the microaerobic dark fermentation is a special case of metabolism, Klamt et al. took a deeper look into the activity of enzymes in the electron transport chain (ETC) of *R. rubrum* [77]. For this purpose, they used readily available information of the fermentation process, such as growth rate, concentration of ATP and NADH, or bacteriochlorophyll (Bchl) and simulated the activity of enzymes of the ETC as differential equations and compared the different states of fermentation. A brief overview of the ETC pathway and the activity under microaerobic dark conditions compared to an aerobic dark growth regiment are depicted in Fig. 6.

During microaerobic dark fermentation, no activity of the reaction complex (RC) in the light harvesting system could be detected, confirming that no energy is generated by light.

The higher level of the NADH concentration cannot only be explained within the metabolic pathways (see above), but also at the level of the ETC. A central aspect of this is the reduction of cytochrome oxidase activity due to oxygen limitation, which additionally reduces Q to QH_2_ and cytochrome c_2_^3+^ to c_2_^2+^. Due to the predominant presence of reduced QH_2_, less substrate is available for the activity of NADH dehydrogenase, which thereby can explain an increased NADH/NAD^+^ ratio. As cytochrome oxidase and ubiquinol oxidase, because of the abundance of QH_2_, are active, an over-reduction of the ETC is prevented by lowering the P/O ratio, as oxygen can still be reduced to water indicated by the drastically increased activity of the ubiquinol oxidase. Due to the generation of a proton-motive force, as oxygen is still available, an activity of ATP synthase is observable resulting in an equal ATP/ADP ratio compared to aerobic, dark growth and photofermentative growth (see Table 3).

**Table 3:** Energy state of the *R. rubrum* model organism during different cultivation conditions [77]

Substrate	Aerobic growth in the dark	Anaerobic growth in low light	Microaerobic growth in the dark
ATP/ADP	2.70 [78]	2.70 [78]	2.70 [78]
NADH/NAD^+^	0.05 [76]	0.71 [76]	0.71 [76]
Bchl [µmol g_CDW_^−1^]	0.05 [20]	8.20 [79]	1.22 [20]
Growth rate µ [h^−1^]	0.15 [20]	0.10 [79]	0.10 [79]

With the additional energy in the form of ATP and the low oxygen tension, an additional nitrogenase activity can be enabled, allowing an additional hydrogen formation in this state. A similar formation of photosynthetic membranes during microaerobic dark fermentation has not yet been observed for *R. palustris* and *R.* *capsulatus* on the modified Sistrom medium used for *R. rubrum* [20]. Therefore, principles of cellular over-reduction during microaerobic dark fermentation are currently only applicable for *R. rubrum;* further research regarding substrate composition, oxygen partial pressure, and redox potential is necessary to evaluate if other purple bacteria could express a similar behavior.

## Challenges of the microaerobic dark fermentation

Out of this overview, two key challenges for microaerobic dark fermentation arise: maintaining the microaerobic state as well as ensuring hydrogen production even at high-cell densities.

### Controlling the microaerobic fermentation state

The most essential challenge is precisely controlling the microaerobic state to induce hydrogen production. Over the years, different methods were conducted and are summarized in Table 4. While clark electrodes are still commonly used in most laboratories, limits are met regarding the sensor sensitivity at low oxygen partial pressures with a lower limit of the linear range that equates to around 3% pO_2_ and a level of detection around 0.3% pO_2_, which is sufficient for PNSB with a higher microaerobic threshold, as *R. palustris* or *R. capsulatus*, but makes it inherently unsuitable for microaerobic conditions, as they are observed for *R. rubrum* [80]. Optical dissolved oxygen sensors can overcome these limits, as they have true linearity down to 0% dissolved oxygen without consuming oxygen and affecting readings, but they are susceptible to pH and temperature and can affect the stability of organic molecules [81].

**Table 4:** Overview of different strategies for controlling the microaerobic state of PNSB

	Advantages	Disadvantages	Sources
pO_2_-detection by sensors	Equipment commonly available	Established clark electrodes offer lower sensitivity	[19]
Control by pH	Easy setup in batch fermentations	Only applicable for batch processes, as feeds alter the pH	[20]
Redox control	High sensitivity even under low oxygen conditions	Redox potential is influenced by substrates and metabolites	[19]
Model-based feedback control	Allows precise control and switch between microaerobic steady states	Currently limited to substrates and oxygen as growth influencing parameters	[82]
Artificial neural networks	Maximize yields by predictive process control of O_2_ supply	Complete understanding of metabolism for metabolic mapping necessary	[83]

pH control was first proposed by Ghosh et al. and Grammel et al. using two fermentation substrates, fructose for glycolysis and succinate for the TCA cycle; microaerobic conditions can be adjusted based on the pH of the fermentation media. While the fermentative consumption of fructose is increased under anaerobic conditions and results in a pH decrease, succinate is mainly metabolized by oxidative metabolism and ensues a rise in pH as NaOH is produced by a succinate: H^+^ symporter [20, 84]. Microaerobic conditions are met when the pH is balanced at a pH of 6.85 and an equilibrium between fructose and succinate consumption is reached, indicated by the production of photosynthetic membranes in the dark and the purple coloration of the bacteria. This concept offers an easy way of maintaining microaerobic conditions by pH-regulated culture aeration and stirring with common fermentation equipment, decreases the consumption of pH correction media, and, therefore, culture dilution. With the drawback of being limited to batch fermentations, feeding during fermentation and metabolite accumulation can alter the pH, which makes the regulation inaccurate. Such a regulation loop has not yet been assessed for other PNSB.

Control by redox signal to control the microaerobic state was established by Carius et al. to observe the metabolism of *R. rubrum* to different levels of redox potential (and, therefore, dissolved O_2_) and consequently developed to stabilize the state in a continuous fermentation and can be applied, where common clark electrodes fail [19, 82]. It was already shown that the redox potential directly depends on the concentration of dissolved oxygen in the fermentation media [85, 86]. However, beside dissolved oxygen, the measured redox potential is influenced by all reducible or oxidizable molecules in the fermentation media [87]. To increase the reliability of the dependence between redox signal and dissolved oxygen, Carius et al. proposed methodic rules. First, to ensure reproducibility, each medium is checked prior to inoculation to ensure that it has a consistent redox potential at the start. Second, the redox potential is not treated as an absolute value but as a difference or delta and is used to achieve a stepwise reduction of the redox potential in a short period of time. This reduces the risk of the measurement being biased by previous fermentation history and changes during the stepwise reduction [19].

This control was later further developed by Carius et al. and expanded by the establishment of a model-based feedback control [82]. This control was developed under two conditions: microbial growth is only dependent on the available substrate and oxygen concentrations. To simplify it, the framework was set that cultivation only takes place at substrate levels that neither act limiting nor inhibiting. After validating model functions, which described growth rates under various oxygen limitations (in the form of stepwise reduction of redox potential) by Hill kinetics, as well as defining and validating correlations, such as individual substrate-related growth rates and uptake rates, a model for continuous microaerobic cultivation could be formulated. This further enabled a new microaerobic control strategy, based on the initial redox potential control, in the form of a model-based two-degree-of-freedom controller. Comprising two parts, a feedforward model-based part that controls the cultivation based on the previously proposed model equations and a PI feedback controller in the form of a biomass probe that minimizes the error between the predicted output by the model and the measured one in a closed loop by adjusting the dilution rate. While it is a more complex method of controlling the microaerobic state in a steady state compared to the others, it enables the precise investigation of microaerobic phenomena in cultures without the limitations associated with a batch process. However, for transfer to other process conditions, it must be ensured that the defined framework conditions are adhered to, as deviations can occur in the model and in the feedback measurement by the biomass probe due to the formation and accumulation of toxic metabolites or increased expression of alternative metabolic pathways, such as the ethylmalonyl metabolic pathway and the associated more dominant formation of PHA [15].

Finally, the most recent advance in microaerobic process regulation represents the use of artificial neural networks (ANN), which are trained using metabolic flux mapping and take control of the fermentation process on the basis of predictive models. This has already been demonstrated using an example of controlling the O_2_ flow of an *S. cerevisiae* alcohol fermentation by Mesquita et al., but has not yet been applied to microaerobic dark fermentation with PNSB [83]. Their approach is based on creating an in silico metabolic mapping of the yeast, in which a training data set for the ANN is created by varying the carbon and oxygen assimilation flows with the aim of maximizing ethanol yield. The trained ANN can then minute by minute evaluate and control the oxygen flux needed in relation to the current metabolic state and need of the cells for an increase in product formation and an overall optimization of microaerobic state control. Using only gas phase composition (CO_2_ and O_2_) without the need of any additional probes, thus increasing yield by up to 37%.

Such in silico mapping of a metabolism appears attractive at first glance, as no experimental work is necessary, but it requires the existence of a complete metabolic model of a microorganism. In the case of a model organism, such as yeast, there is a complete metabolic model, such as the iND750 GMS model from Duarte et al., but none for PNSB yet [88]. This process is generally applicable to microaerobic dark fermentation as well but currently still requires either scientific progress in designing a metabolic model, or the experimental, manual labor-intensive preparation of a training data set, but offers a highly interesting perspective for future research improving microaerobic state control.

### Understanding how media composition will influence microaerobic dark fermentation

The topic of microaerobic fermentation is still fairly new and has started to gain traction only in the last 10–15 years. There are different opinions regarding the influence of substrate and oxygen on hydrogen production under microaerobic conditions. Autenrieth et al. and Selao et al. interpret the microaerobic regiment as an over-reduction of *R. rubrum* by the production of excess reduction equivalents during a partly anaerobic metabolism of fructose or pyruvate resulting in hydrogen evolution by the PFL pathway and/or by nitrogenase depending on the ammonium concentration present [6, 8]. Meanwhile, Hallenbeck et al. proposed that there is never complete substrate utilization of organic acids under anaerobic conditions. For example, during the metabolization of lactic acid, a theoretical maximum of 33% substrate conversion can be reached. As lactate is metabolized to acetate (∆G^0’^ = − 4.2 kJ) but can then not be further metabolized as a huge amount of free energy (∆G^0’^ =  + 104.6 kJ) is required to continue the metabolism [9]. The approach of Hallenbeck et al. follows the idea that the necessary energy for complete substrate metabolism can be provided by allowing oxygen conditions that are not too inhibitory for nitrogenases but still supply enough oxygen for oxidative phosphorylation and was shown for *R. capsulatus* and *R. palustris*. The ATP generated can ensure a high efficiency of substrate conversion as well as provide energy for nitrogenase for hydrogen production, which would otherwise be provided by lighting, making the nitrogenase pathway the main route for hydrogen formation.

Both can be explained by the microaerobic growth regiment (as determined by amounts of cytochrome oxidase present) of the PNSB as it is vastly different from high possible oxygen values for *R. capsulatus* (pO_2_ = 4–8%) and *R. palustris* (9%) (confirmed by Hallenbeck et al.) and extremely low for *R. rubrum* (≤ 0.3%) (determined by Autenrieth et al. and Selao et al.) [6, 8–10]. A conclusion could be drawn that the higher oxygen levels in *R. palustris* and *R.* *capsulatus* enable a significantly higher activity of the TCA cycle and, therefore, a higher substrate utilization of organic acids (which has not been shown yet) and thus also of oxidative phosphorylation compared to *R. rubrum*. This enables more efficient energy production and higher nitrogenase activity. The hypothesis that H_2_ is mainly produced by the PFL pathway is backed by the accumulation of organic acids shown in the microaerobic fermentation of *R. rubrum*, which could explain why *R. rubrum* relies on two substrates and requires an additional reduction by glycolysis of sugars for hydrogen production [19]. As already mentioned, *R. rubrum* possesses the ability to synthesize the photosynthetic membranes even in the dark, which was not identified yet for other PNSB. The question arises if *R.* *capsulatus* and *R.* *palustris*, mainly producing H_2_ by nitrogenase, need a photosynthetic seed culture as a H_2_ production condition or if nitrogenase can be expressed in other ways. This would result in a loss of the many benefits of the dark fermentation regime, such as the lack of necessity for photoreactors and a simple fermentation method. One of the key factors for enabling H_2_ production with *R. capsulatus* and *R. palustris* will be a deeper understanding of the formation of photosynthetic membrane and the nitrogenase if a strictly dark fermentation process is conducted.

### Quorum sensing

Quorum sensing is the communication between bacteria via chemical signal molecules. These signaling molecules are formed as a function of fluctuations in the cell density of the bacterial culture. The gene expression of bacterial proteins is regulated by those signal molecules, which is generally intended to help the bacteria cope with the changed environmental conditions. Research in this context is primarily concerned with the biofilm formation of pathogens, such as *Streptococcus sp*., but quorum sensing behavior has also been observed in PNSB [31, 89]. At a high-cell density (starting around 15 g L^−1^ CDW), the ability of *R. rubrum* to form photosynthetic membranes during microaerobic dark fermentation and thus also the activity of nitrogenases is reduced. This mechanism is presumably there to prevent inefficient photofermentation due to the self-shading of the bacteria in high-cell densities. This is triggered by the formation of so-called N-acetylated homoserine lactones (AHL). As higher cell densities are associated with an increased hydrogen production rate, quorum sensing is one of the main obstacles for increased productivity. Although purple bacteria can be cultivated at exceptionally high-cell densities, the advantages of microaerobic dark fermentation are lost due to quorum sensing. To overcome this, either quorum quenching could be performed or adapting the strategy of fermentation. Quorum quenching can be induced by different strategies using inhibitors that interfere with the production or recognition of AHLs, by binding through antibodies or hydrolysis by enzymes [90]. All of which are highly cost-demanding, making a transition of fermentation strategy into continuous fermentation with a steady microaerobic state, as researched by Carius et al. or the use of membrane bioreactors with cell retention to “wash out” AHLs attractive. [82].

### Purification of fermentative biohydrogen

Physical purification of hydrogen from gaseous mixtures is mainly divided into three technologies: adsorption (pressure swing adsorption, temperature swing adsorption and vacuum swing adsorption), low-temperature separation (cryogenic distillation and low-temperature adsorption) and membrane separation (with inorganic or organic membranes) [91]. While adsorption and low-temperature separation is well-established on a larger, industrial scale both processes require high energy consumption, especially if the concentration of the product stream is comparably low. For these reasons, the recent research on purification of hydrogen from biotechnological fermentation focused on the separation using membranes as they require minimal energy, can purify even low concentrated product streams and still ensure a high product purity. Downsides are a high cost if, for example, palladium membranes are used and a low selectivity when using organic membranes. Inorganic membrane materials exhibit a better separation performance, e.g., higher separation efficiency, but can have problems with long-term stability and require more energy, metallic membrane materials can overcome problems like the brittleness but require high temperatures and higher pressures for a high separation performance (400–500 °C and 1–5 bar) [92–94].

To transfer the hydrogen purification process to fermentative biohydrogen production, the scenarios must first be defined and disruptive factors in the exhaust gas for purification identified. The first starting point is an exhaust gas composition only consisting of the products of dark fermentation—CO_2_ and H_2_. This mixture can be separated using either H_2_ or CO_2_ selective membranes, a process that has been well-researched. H₂ selective membranes can be organic polymer-based, made of polyimides or polybenzimidazoles, or inorganic, made of ceramics, palladium, zeolites, graphene, or graphene oxide [95]. CO_2_ separation is particularly achieved using polyethylene oxide-based membranes due to the high affinity between CO_2_ and ethylene oxide [96].

On the basis of these findings, the scenario needs to be expanded, as the exhaust gas naturally contains not only the products of the dark fermentation, but also a large proportion of nitrogen, since a bioreactor system needs to be gassed with nitrogen to create microaerobic or anaerobic conditions. Nitrogen is also the educt for the photofermentation reaction and is, therefore, necessary for H_2_ production. Such an exhaust composition can be expected to contain approximately 10% H_2_, 10% CO_2_, and 80% N_2_. Different membrane materials with regard to their performance at ambient conditions and with low starting concentrations such as 10% H_2_ were evaluated in the literature to simulate conditions that are comparable to the fermentation exhaust gas. To make H_2_ purification economically viable, it makes most sense to establish a membrane separation process with membranes, which does not require high pressures or temperatures but operates at or near conditions of the fermentation process around 30 °C and a pressure between 1 and 2 bar. The concepts are trying to continuously separate the formed H_2_ inline at the bioreactor. Separation selectivity between hydrogen and carbon dioxide depends on temperature, pressure, and membrane material as well as the stream composition. Even at low H_2_ concentration, a separation selectivity of 3.4–5.8 for polydimethylsiloxane membranes and 1.7 for SAPO 34, a zeolith-based membrane, was reached with such an exhaust composition [97–99].

However, since O_2_ is also required for the microaerobic condition to control and maintain this state, the scenario needs to be extended once again. Ideally, in microaerobic dark fermentation, the bioreactor would only be gassed with pure O_2_ at a rate that corresponds exactly to the necessary oxygen uptake rate of the microaerobic condition. The O_2_ is completely consumed, and only the products H_2_ and CO_2_ accumulate in the exhaust air. In reality, however, due to a low O_2_ solubility and a subsequent low concentration gradient, the diffusion forces are small; in addition, distribution problems of O_2_ in the fermentation liquid and a limited residence time of bubbles in the fermentation medium have to be considered as well [100]. Therefore, in practice, gassing must be carried out with a gas quantity that is significantly higher than the consumption rate of the bacteria. Since fermentation processes are usually gassed with compressed air, this realistic scenario results in an exhaust gas composition consisting of N_2_, O_2_, CO_2_, and H_2_. This results in two challenges: the separation of this complex fermentation exhaust gas and the consideration of safety-relevant aspects during fermentation with regard to exhaust gases with a flammable or explosive gas mixture.

The purification with this gas mixture is not widely investigated yet, since anaerobic, fermentative hydrogen production remains the focus of research, where the presence of O_2_ is, therefore, not considered. When using PDMS membranes, problems in separation could arise, as oxygen (~ 600 barrer) and hydrogen (~ 570 barrer) share a quite similar permeability, making separation much more challenging [97, 101]. Palladium membranes could overcome this problem as they are exceptionally selective to hydrogen (α_H2/N2_ =  > 1000) and offer a high permeability, but again, as mentioned above, lack economic viability [102, 103]. To address this issue, research is currently focusing on investigating palladium metal composite materials to reduce costs. For example, Omidifar et al. were able to achieve a cost reduction of up to 38% using a palladium–nickel mixture without any loss of selectivity [104]. Other recently developed methods include the use of highly selective metal–organic framework membranes, which offer high selectivity (α_H2/N2_ = 209) by constructing a zeolitic imidazolate framework membrane with a pore aperture size (3.0–3.4 Å) that allows H_2_ (kinetic diameter of 2.9 Å) to pass through, while O_2_ (3.5 Å), N_2_ (3.6 Å), and CO_2_ (3.3 Å) are retained [105, 106]. However, newer approaches still need to be investigated in terms of their long-term stability and suitability for industrial application. A further development of this is mixed matrix membranes (MMM), in which polymer membranes are equipped with a filler, such as zeolites or a metallic organic framework, to increase the selectivity (α_H2/N2_ =  ~ 10–60) and permeability of the membranes while keeping membrane costs low [107].

H_2_ as a product in an exhaust gas stream in which O_2_ is also present due to the microaerobic conditions poses a safety risk. This is due to the very high explosion range (under normal conditions 20 °C, 1 bar) of 4.1 to 75.6 mol% in air. Based on the chemsafe database it is recommended to limit O_2_ concentration to a maximum of 4.3 mol% or H_2_ concentration to 5.5 mol% for operation without explosion risk [108]. With regard to the safety of operating a fermenter with a potentially explosive mixture, there are two ways of designing the process. On one hand, the process can be run under regulated conditions with an exhaust gas composition in the explosive range. An example for such a regulation is the European Union Directive 94/9/EC (ATEX). When explosive atmospheres occur in processes, measures must be taken to protect employees. Based on these directives, a risk assessment and an explosion protection document must be prepared. This includes, for example, classification into safety zones according to the frequency of occurrence of such atmospheres. In addition, equipment must be approved for operation under these conditions and may only be operated if the maximum surface temperature of the equipment is below the ignition temperature of the gas mixture. The removal of ignition sources and the need for alarm systems must also be considered. On the other hand, an approach can be taken to operate the exhaust gas concentration below the maximum concentrations of H_2_ and O_2_ by increasing the gassing rate or modifying the supply air composition. The pros and cons of both approaches still need to be investigated, as meeting regulatory requirements can result in additional equipment and bureaucratic costs, but reducing the H_2_ concentration in the exhaust gas leads to more complex product purification and the generation of costs due to the gas consumption of diluents, such as N_2_.

## Potentials for increasing hydrogen converting efficiency

Despite challenges to overcome, the microaerobic dark fermentation offers a wide range of advantages, making it interesting for further research. The broad spectrum of fermentation substrates combined with a high resistance towards heavy metals compared to, for example, *E. coli* make it in particular interesting for cultivation on different complex industrial waste streams, such as sugar, organic acid, or alcohol waste from the chemical and agricultural industry [109–111].

Thus, the main challenges in the implementation of microaerobic hydrogen fermentation are the targeted control of the microaerobic state, the achievement of high-cell densities with simultaneous low effects of quorum sensing, and the maximization of H_2_ evolution in the dark.

### Genetic engineering

One promising approach to achieve a substantial increase in the hydrogen yield is the genetic modification of purple bacteria. As most hydrogen-producing bacteria not only have hydrogen-producing enzymes, such as formate hydrogen lyase or nitrogenase but also hydrogen-consuming ones: membrane-bound enzymes called uptake hydrogenases (hup). This enzyme offers the bacteria a pathway to oxidize hydrogen and recover energy in the form of electrons and protons from dissolved hydrogen in the cultivation media [112]:

$$H_{2} \mathop{\longrightarrow}\limits^{Uptake\,hydrogenase}2H^{ + } + 2e^{ - } \to cell\,metabolism\,\,\Delta G^{0} = + 38\,\,kJ \, mol^{ - 1}$$

While this is a strategy to improve the energy efficiency of the bacteria, it can greatly impact the yield of hydrogen for biotechnological processes. Hup-deficient strains of *R. rubrum*, *R.* *sphaeroides*, and *R. capsulatus* were already engineered, resulting in improvements in an increase of hydrogen production rates of 11–30%, considering that the processes were carried out as photofermentation [113–115]. The hup assembly relies on the expression of three genes, *hupS*, *hupL*, and *hupC*, which contain the genetic information for the subunits of the enzyme [116].

Recently, however, research into genetic modifications has expanded into other areas, in particular metabolic engineering, in which metabolic pathways and enzyme expression as well as their respective activities are specifically influenced to focus substrate conversion to H_2_. One option that was pursued by Shimizu et al. was the inhibition of competing metabolism, in this case PHA production, which represents a competing, thermodynamically more favorable product than H_2_ [117]. By subsequently deleting the *phaC1* gene, responsible for PHA synthesis, along with *phaR*, a transcriptional regulator for sensing PHA accumulation, the PHA content of the biomass when grown on acetate under photofermentative conditions could be reduced to one-third. By shutting down this competing metabolic pathway, the H_2_ yield could be increased by up to 40%. An approach that could also find appeal in microaerobic dark fermentation, as PHA contents of up to 30% were already reported [15]. Approaches to further increase H_2_ production by modification of the switch-off of nitrogenase have been developed, for example, by Gao et al., deleting the NifA gene, which is an activator of nitrogenase expression responsible for the ammonium-dependent switch-off effect of nitrogenase in *R. capsulatus* [118, 119]. By deleting the responsible N-terminal domain *nifA1* and *nifA2*, a mutant was developed with a 1.3 to 5.2 fold increase in H_2_ production compared to the wild strain under varying ammonia concentrations during photofermentation. A more promising modification under microaerobic conditions would be to eliminate O_2_ sensitivity of nitrogenase, but this has not yet been achieved. The cause is that the inactivity of nitrogenase is not due to ADP-ribosylation but a consequence of various environmental influences [120]. On one hand, particularly high O_2_ levels irreversibly damage the [4Fe–4S] cluster of the Fe protein in nitrogenase [121]. On the other hand, the electron flow is simply reversibly diverted to O_2_ as the terminal electron acceptor, which also renders nitrogenase inactive [122]. This makes it difficult to increase O_2_ tolerance, even from a genetic engineering perspective. One approach would be to modify the Fd or Fld (flavodoxin) enzymes, or their reduction, with the aim of providing low-potential electrons even in oxygen environments, which is necessary for the nitrogenase-catalyzed reaction [123].

### Increasing activity of nitrogenase by switch-on regulation as well as promoting formate hydrogen lyase pathways

The first potential to increase product yield that comes to mind is the reactivation of nitrogenase enabling simultaneous H_2_ production together with the PFL pathway. The two most prominent parameters to be considered (see Fig. 3) are the choice and concentration of nitrogen and the energy condition of the cell.

Switch-off of nitrogenase is often associated with an abundance of ammonium but can also be induced by glutamate as a nitrogen source. The effect can be explained by the absence of necessity. If a source of nitrogen is present in the media, an activity of nitrogenase for nitrogen fixation is not necessary and probably switched-off for energy servings of the cell. Influence of nitrogen sources was mainly investigated by Hallenbeck et al. for *R. capsulatus* [10, 124]. Hydrogen productivity was maximized at an ammonium sulfate concentration of 4 mM and a concentration of 2–4 mM for sodium glutamate, respectively. In this case, ammonium generally had a more negative effect, only reaching around 70% of productivity compared to glutamate and completely inhibiting any activity at 6 mM ammonium. When measuring nitrogenase activity, it was apparent that only a tenth of the activity of photofermentative conditions could be reached during microaerobic fermentation, based on a lack of sufficient ATP generation, therefore, creating a new potential for increasing hydrogen productivity by providing optimal culture conditions. A deeper understanding was achieved when cultures were subjected to different ammonium concentrations, observing differences in nitrogenase sensitivity to light and ammonium addition depending on if the culture was cultivated with or without ammonium during the seed culture phase. Interestingly, cultures that were previously adapted to ammonium during previous cultivations exhibit a delayed reduction of nitrogenase activity and a lower ribosylation of nitrogenase when subjected to darkness and a higher tolerance for high concentrations (40 mM) of ammonium, responding with a magnitude response instead of a total switch-off, with a complete recovery of activity in 25 min. These results imply that preconditioning cultures with ammonium or limiting ammonium during certain fermentation phases could be a key factor for high activities of nitrogenase under microaerobic conditions.

Another approach to reactivate nitrogenase activity was first discovered by Schneider et al. and Ludden & Burris and further characterized by Selao et al. using pyruvate as a substrate [6, 73, 125]. The effect of pyruvate on nitrogenase was initially observed for *Chromatium vinosum* by Yoch and Arnon and later adapted for *R. rubrum* [126]. The mechanism is not completely understood yet, but the main hypothesis, as suggested by different authors, is that pyruvate acts as an electron donor for the nitrogenase reaction, which was also observed for α-ketoglutarate and oxalacetate. While other substrates such as formate, succinate, or malate can also act as electron donors, no nitrogenase activity was observed. It was concluded that the metabolism of these substrates to pyruvate consumes the pool of available ATP, simply using up the substrate of the nitrogenase reaction. Later on, Selao et al. gained a deeper insight into the ribosylation of nitrogenase and drew this conclusion: ATP/ADP ratio and the activity of pyruvate formate lyase, and therefore, the PFL pathway is necessary for nitrogenase activity, as nitrogenase is switched-off at lower ratios. Therefore, the addition of pyruvate can act as a donor substrate. PFL can provide ATP by conversion of pyruvate to acetyl-CoA and subsequently acetyl-P, which then can generate ATP and acetate by acetate kinase. If we combine the need for high ATP levels and the presence of precisely these (see Table 3) during microaerobic dark fermentation, a similar effect without the need for pyruvate could be achieved. This results in two approaches that can be pursued to further increase nitrogenase activity: the implementation of nitrogen limitation at the beginning of the production phase and the provision of sufficient energy in the form of ATP, either through the choice of substrates or controlled microaerobic cultivation. If the same level of ATP can be obtained with other substrates is still a necessary field of research for enabling an additional H_2_ production on various substrates and waste streams.

Another approach to increasing the molar H_2_ yield, besides regenerating nitrogenase activity, is to investigate possibilities for increasing the second production pathway, dark fermentation via formate hydrogen lyase. As this pathway also offers quite a lot of possibilities for improvement, only yielding between 0.4 to 1 mol H_2_ mol^−1^ substrate under anaerobic and 0.2 to 1.4 mol H_2_ mol^−1^ substrate under microaerobic conditions compared to the theoretical maximum yield of 4 mol H_2_ mol^−1^ substrate [8, 9, 33, 127]. As most literature focuses on the photofermentative capabilities of these strains, research into dark fermentation is still very limited and requires further expansion. Therefore, the following description includes methods for improving yields for dark fermentation by facultative anaerobes in general and still needs to be verified for the use case of microaerobic conditions. There are enzyme-specific possibilities: pH, temperature and protein expression. Control of the pH is a central factor influencing not only FHL activity, by providing the specific pH optimum, but also the enzyme activity of other metabolic pathways indirectly influencing H_2_ evolution. In the case of *R. sphaeroides* dark fermentation a four time increase in yield was reported by a shift of pH from 6.5 to 8.0 [128]. Same effects were apparent regarding cultivation temperature, an increase in temperature in the range of the temperature optimum can increase the yield, as higher temperatures increases the entropy and the free energy of the system which makes H_2_ production thermodynamically more viable. This effect is especially expressed in thermophiles [61]. Another leverage is the degree of expression of the enzyme itself, which is especially dependent on a regulator protein called FhlA. Increasing FhlA expression by genetic engineering resulted in a 39% increase in yield in the case of *E. aerogenes* [129]. This is especially relevant in the case of microaerobic cultivation, as FhlA expression was found to be oxygen dependent and decreasing at higher oxygen concentrations, which needs to be subject of further research [130, 131]. Finally, a complete oxidation of substrates and the regulation of the flow of donated electrons offer the highest potential, which is not only applicable in dark fermentation but also photofermentation. As most electrons are not transferred to H_2_ but to organic acids such as acetate, propionate, formate or succinate or to competing electron sinks, such as PHB or CO_2_ fixation, and can be controlled by genetic engineering or precise control of the microaerobic state [15, 19, 67].

### H_2_ vs. PHB production—competing electron sinks

**Fig. 7:**
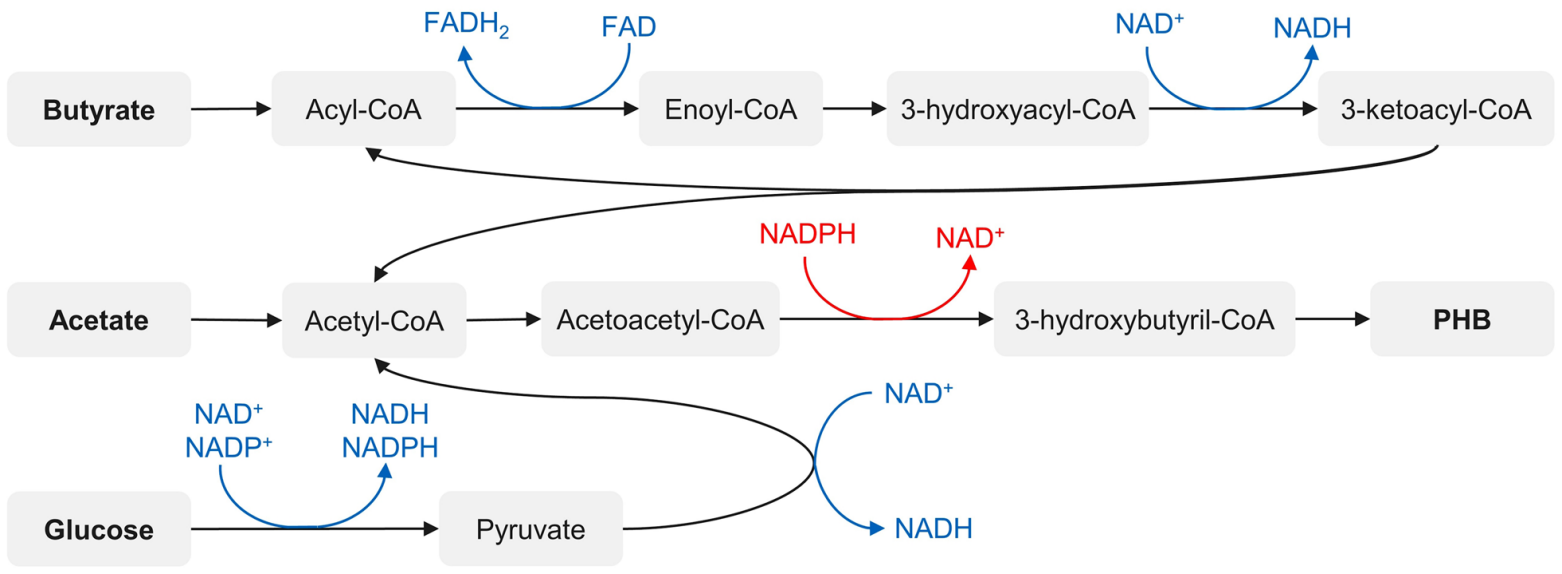
Synthesis of PHB from butyrate, acetate, and glucose. Electron sink reactions are shown in red, electron donor reactions are shown in blue. Pathways proposed from Blunt et al. [134]

Changes in the oxygen state of all bacteria in general, but especially in the PNSB, can disturb the redox balance of the bacteria. To counteract this, there are a number of redox-balancing mechanisms, which are summarized under the term redox homeostasis that can act as so-called electron sinks. Electron sinks are necessary to get energy from organic substrates by accepting electrons and, therefore, enabling chemical reactions. So far, the fixation of CO_2_ and N_2_ as well as the production of H_2_ and PHB were observed as electron sinking strategies for nearly or strict anaerobic cultivation of PNSB. When subjected to microaerobic conditions up to 30% of PHBV could be observed in the case of *R. rubrum* [15]. The fact that hydrogen production competes with other strategies makes this potential possibly the one with the highest reward if H_2_ production could be established as the primary used electron sink by control of metabolism, process conditions or by genetic engineering. The main obstacle as called by researchers is the ‘thermodynamic hierarchy’ of redox strategies, e.g., how thermodynamically favorable a redox strategy is. The hierarchy starts with PHB, which has the thermodynamically lowest requirements (+ 86–133 kJ mol^−1^ C_biomass_), followed by CO_2_ fixation by Calvin cycle (+ 98–144 kJ mol^−1^ C_biomass_), which is mostly observable in anaerobic but also partly in microaerobic cultivation regiments, and finally N_2_-fixation with H_2_ as byproduct is chosen by the PNSB as the nitrogenase reaction is highly energy-intensive (+ 274 kJ mol^−1^ C_biomass_) [67, 132]. Even though PFL pathway seems thermodynamically very favorable, this hierarchy does not affect H_2_ evolution by formate hydrogen lyase as it is not acting as an electron sink, providing electrons for reaction from formate (see above). One way to influence the hierarchy is the choice of substrate. For instance, during acetate assimilation as a sole substrate, PHB can act as an electron sink, since NADH is reduced during PHB synthesis and two electrons are accepted (see number of sink (red) and donor (blue) reactions in Fig. 7). This is not the case for substrates such as butyrate, propionate or glucose as more electrons are donated during β-oxidation, respectively, glycolysis than by reduction of NADPH [55, 133].

This implies a strong argument for the use of substrates that do not act as electron sinks by PHB production to bypass the thermodynamic hierarchy and to increase product yield of hydrogen per substrate but need to be further tested in a microaerobic fermentation regiment as such effects of substrates have not been documented yet. To address the second problem in the thermodynamic hierarchy, CO_2_-fixation, there are also approaches already being investigated in relation to dark fermentation or photofermentation. Two approaches are currently being pursued: on one hand, the removal of CO_2_ in the medium and in the reactor headspace by gassing control to limit the amount of CO_2_ available for fixation [135]. On the other hand, the use of genetically engineered deletion strains is being investigated, in which the function of the Calvin cycle for CO_2_ fixation is prevented [60]. Both approaches have led to substantial increases in H_2_ yield. Whether these approaches are also promising under microaerobic conditions has not yet been investigated. The unclear situation as to whether the Calvin cycle is active under microaerobic conditions is likely to be particularly relevant here, as a still active Calvin cycle would offer room for major yield improvements by disabling it.

### Industrial competitiveness, economic considerations and opportunities

Considering the current state of research, it is already possible to estimate the competitiveness and production costs of an industrial plant; attempts to do so have already been made by Full et al. and Gamero et al. [136, 137]. The simulated process design was based to a larger extent on dark fermentation, which shares many identical steps (apart from process control and gas composition). To establish an economical process, a biorefinery concept was used in both cases, resulting in high economic efficiency through the use of waste streams as substrate, the material and energy integration into a plant concept, and the complete financial utilization of all byproducts. First proposed model was the concept of hydrogen bioenergy with carbon capture and storage (HyBECCS) exploring the capability of converting fruit wastes to hydrogen by microaerobic dark fermentation with the additional storage of CO_2_ after gas separation forming a negative emission process at a scale of common biogas plants (6000 m^3^). Based on small scale estimations with yields currently achievable a simulated cost of 4.00 EUR kg^−1^ H_2_ was calculated, which does seem to indicate to be competitive with other green hydrogen technologies, such as electrolysis from renewable energy (5.67 EUR kg^−1^ H_2_). The plant is expected to have an initial capex of 10 million EUR, 27% of the costs are allocated for the fermentation process, 7% for the bioreactor. When including green house gas internalization by carbon capture and storage this can be decreased to just 0.43 EUR kg^−1^ H_2_. However, these calculation comes with some limitations, as it assumes a price of 195 EUR tCO_2_eq^−1^, which represents the actual cost of climate damage, but is considerably lower than the current pricing in Germany (2025: 55 EUR tCO_2_eq^−1^), artificially decreasing the simulated price [138]. An increased H_2_ yield was proven to be one of the most impactful, promising levers for further research, reducing costs by approximately 0.6 EUR kg^−1^ H_2_ at a 10% increase. While this simulation focuses primarily on economic evaluation, consideration of another biorefinery concept also highlights the challenges that still need to be overcome for industrial application of microaerobic dark fermentation. In this concept a full utilization of byproducts, such as proteins for animal feed from biomass and the extraction of carotenoids was considered, while the process is coupled with algae cultivation for the biological fixation of the CO_2_ produced during fermentation. After conducting an LCA two main challenges that need to be tackled in the future become apparent: first, the energy consumed during the fermentation in form of H_2_ is threefold more (419 MJ kg^−1^ H_2_) than the energy generated (120 MJ kg^−1^ H_2_), leaving the microaerobic fermentation currently energetically non-self-sufficient. While for dark fermentation a near net positive energy balance is already achievable in some cases [139, 140]. Second, the simulated biorefinery generates 24.4 kgCO_2_eq^−1^ kg^−1^ H_2_, which is substantive higher than the emissions from steam reforming (12.3 kgCO_2_eq^−1^ kg^−1^ H_2_); meanwhile, just 0.7% of the CO_2_ is produced biogenic. It should be noted that the calculations are based on the current electricity mix in Germany (2024) and do not consider recycling of material or energy. Under the same condition even electrolysis is hardly competitive (23.3–32.1 kgCO_2_eq^−1^ kg^−1^ H_2_) [141, 142]. This results in environmental impact as a second hurdle alongside price. To design the process to be competitive from an ecological perspective, a more in-depth intensification and optimization of the biorefinery concept with regard to energy management is necessary. Furthermore, H_2_ yield is identified as a key factor again in a sensitivity analysis, whereby a yield of 0.6 mol H_2_ mol^−1^ fructose halves the CO_2_ equivalents already. Nevertheless, it could be shown that with a realistic increase in H_2_ yield, microaerobic dark fermentation can already be implemented in a way that is industrially competitive today.

## Conclusion

The need to diversify hydrogen production is becoming increasingly urgent. Extensive research has been carried out on biotechnological hydrogen from dark or photofermentation, but it is not yet industrially viable. To advance this goal, the use of waste streams from the food and chemical industry to close energy and material cycles is getting increasing attention. However, it is also worthwhile to explore new concepts in the field of biotechnological hydrogen production. One exciting new perspective is presented in this review: the microaerobic dark fermentation. Admittedly, the research is still at an early stage and the results are still in their infancy, but there is great potential and promise that, with further research, a new chapter in fermentative hydrogen production could be opened. The combination of aerobic and microaerobic cultivation of PNSB offers high growth rates, cell densities, and can enable the efficient conversion of substrates to hydrogen through the utilization of two production routes.

Since the microaerobic H_2_ production process currently still lags behind or is only comparable to the efficiencies of dark fermentation, three main measures are proposed at the end of this review that could be prioritized in further research: adjustments regarding quorum sensing, an increase in substrate utilization efficiency, and increased reactivation of nitrogenase activity. Quorum sensing is relevant as it limits a main advantage of facultative anaerobes, the implementation of a high-cell-density process without losses in product yield, increasing H_2_ production rates per volume of reactor as well as space–time yields. Possible measures would either be to introduce quorum sensing inhibitors, some of which are costly, or to genetically modify the strains to suppress the expression of AHLs producing pathways. Increases in substrate utilization efficiency are necessary; microaerobic dark fermentation itself should already be a solution to this problem as it allows partial activity of the TCA cycle, but still a surplus of organic acids was generated in the literature implying overflow metabolism, which lowers conversion efficiency and prevents extended production times by reaching toxic levels [15, 19]. Solutions could either take the form of adapting feeding strategies to inhibit overflow metabolism or, as demonstrated by Sagir et al., converting metabolites in a sequential photofermentation step, which, in turn, comes with the disadvantage of losing the benefits of the dark process but promotes substrate conversion to H_2_ [74]. The most important aspect, the regeneration of nitrogenase activity, should be a particular focus, as it represents the greatest leverage for increased yields. Approaches could be either the implementation of measures to reduce the impact of switch-off factors, such as ammonia limitations during fermentation when entering the microaerobic condition. Or too deepen the understanding how cellular energy levels (in form of ATP) can be controlled on a metabolic level, as Selao et al. identified ATP availability as one of the main factors [6].

In order for microaerobic dark fermentation to be relevant for industrial transfer, it must first exceed the yields of dark fermentation and measure up to the yields of photofermentation. Second, a long-term production phase of several weeks must be made possible, as previous research has been limited to a few hours to a few days, and the long-term stability of nitrogenase activity must be proven as well. Third, transfer to waste materials from the food or chemical industry should be considered to keep production costs low. If these problems are addressed, the new concept of microaerobic dark fermentation could enable a high yield H_2_ production in a quite simple setup, only requiring common bioreactors without complex lighting, tackling current problems of biotechnological H_2_ production and contributing to meeting the increasing hydrogen demand in the future.

## Data Availability

Not applicable.

## Abbreviations

AHL: N-acetylated homoserine lactones
ANN: Artificial neural network
Bchl: Bacteriochlorophyll
CDW: Cell dry weight
CSTR: Continuous stirred-tank reactor
DF: Dark fermentation
DRAT: ADP-ribosylation transferase
EMP: Embden–Meyerhof–Parnas pathway
ETC: Electron transport chain
ETM: Ethylmalonyl pathway
FDH: Formate dehydrogenase
FHL: Formate hydrogen lyase
MDF: Microaerobic dark fermentation
QH_2_: Ubiquinol
PF: Photofermentation
PFL: Pyruvate formate lyase
PFOR: Pyruvate ferredoxin oxidoreductase
PNSB: Purple noon-sulfur bacteria
PHB: Polyhydroxybutyrate
PHBV: Poly(3-hydroxybutyrate-co-3-hydroxyvalerate)
pO_2_: Oxygen partial pressure
RQH_2_: Rhodoquinon
RuBisCo: Ribulose bisphosphate carboxylase
TCA: Tricarboxylic acid cycle
WoS: Web of Science
